# A draft genome of *Steinernema diaprepesi*

**DOI:** 10.21307/jofnem-2020-069

**Published:** 2020-07-17

**Authors:** Anil Baniya, Jose C. Huguet-Tapia, Peter DiGennaro

**Affiliations:** Department of Entomology and Nematology, University of Florida, Gainesville FL, 32611

**Keywords:** Entomopathogenic nematode, Biological control, Genomics

## Abstract

Entomopathogenic nematodes within the genus *Steinernema* are used as biological control agents against significant agricultural pests. *Steinernema diaprepesi* is native to Florida and very effective in controlling citrus root weevil, a devastating pest of citrus, ornamental plants, and vegetables. Here, we present the draft genome of *Steinernema diaprepesi*, which is a valuable tool for understanding the efficacy of this nematode as a biological control agent.

Citrus root weevil, *Diaprepes abbreviates*, is an important pest of citrus, ornamental plants, and other vegetables in Florida and is spreading throughout southern Texas and southern California (Lapointe et al., 2007; Stuart et al., 2008; Cherry et al., 2011). Root weevil is polyphagous, its increasing geographical distribution has made them a subject of quarantine and eradication programs (Stuart et al., 2008; Campos-Herrera et al., 2015). Soil-applied halogenated hydrocarbons are effective against this insect, but have since been deregistered. Currently, there are no effective registered pesticides against this insect pest (Campos-Herrera et al., 2015). Studies on the use of biological control agent to manage this weevil has been of interest for the past couple of decades (Beavers et al., 1983; Shapiro et al., 2000) and as a result, the use of entomopathogenic nematodes as control agents was found to be effective on *D. abbreviates* larva (McCoy et al., 2002; Ali et al., 2010; Duncan et al., 2013). Entomopathogenic nematodes endemic to citrus growing regions in Florida include *Steinernema diaprepesi* and *S. khuongi* and their role in determining the distribution of root weevil is also evident (Nguyen and Duncan, 2002; Duncan et al., 2003; Stuart et al., 2008; Campos-Herrera et al., 2013; Stock et al., 2018).

The endemic entomopathogenic nematode *S. diaprepesi* is commercially applied to control citrus root weevil. As an obligate parasite, *S. diaprepesi* relies on the toxin produced by its symbiotic bacteria *Xenorhabdus doucetiae* to kill insect hosts (Goodrich-Blair and Clarke, 2007; Stock and Blair, 2008; Castillo et al., 2011). The genome of any organism is the basis of the biological, molecular, and cellular processes that are vital for development and reproduction as it encodes the entire inheritance message of living organisms. Improved understanding of the genome aid in the knowledge of complex gene networks, molecular mechanisms of underpinning symbiosis and pathogenicity, and also provides a foundation for engineering trait improvements (Bolger, Weisshaar, Scholz, Stein, Usadel and Mayer, 2014; Lu et al., 2016; Rodríguez-Leal et al., 2017). The full genome sequence of *X. doucetiae* is currently available and provides a resource for understanding the evolution of virulence genes in bacteria. However, there is little information about the genome of the nematode (Ogier et al., 2014).

In this study, the genome of *S. diaprepesi* was sequenced and assembled. This information will be very valuable to understand the mechanism of evolution, molecular processes that determine parasitism and symbiosis within this complex system followed by the genetic features that make this nematode more effective against citrus root weevil and the extent of their host range.

Nematode samples for genome sequencing were received from Dr. Duncan’s lab at UF/IFAS Citrus Research and Education Center. To confirm the identity of nematodes, we sequenced the ITS region of the ribosomal DNA. The primers used were AB28: 5´-ATATGCTTAAGTTCAGCGGGT-3´ and TW81: 5´-GTTTCCGTAGGTGAACCTGC-3´ The protocol for DNA extraction and condition for Polymerase chain reaction (PCR) amplification (reaction and cycling condition) were followed as Hominick et al. (1997) (Stock et al., 2018). The sequences were queried at the NCBI nucleotide database utilizing megablast with other sequences available at the GenBank using the BLASTn similarity search program. Finally, nematode was confirmed as *S. diaprepesi* with 99.07% identity. Approximately 10,000 freshly collected infective juveniles (IJ) were surface sterilized. Sterilized nematodes were flash-frozen in Liquid nitrogen and thawed twice for DNA extraction. High molecular weight genomic DNA was extracted using a phenol-chloroform method (Donn et al., 2008). The DNA pellet was resuspended in 100 μl Tris-EDTA buffer. University of Florida’s campus-wide Interdisciplinary Center for Biotechnology Research (ICBR) NextGen DNA Sequencing Core Facility (Gainesville, FL) performed library preparation and sequencing using MiSeq Illumina sequencing platform with 2X300v3 format.

A total of ~22 million reads were generated, comprising 6.46 Gb using 300 bp paired-end sequencing. The sequence quality of the raw reads was analyzed using FastQC (Andrews, 2010). Quality trimming, read filtering, and removing adapter contamination were performed using Trimmomatic/0.36 (Bolger, Lohse and Usadel, 2014). Clean reads were subjected to De Novo assembly using the SPAdes/3.13.0 assembler (Bankevich et al., 2012) with Kmer size of 21, 33, 55, 77, 99, and 127. Assembly obtained from kmer 127 was used for downstream evaluation based on fewer contigs and higher N50. Preliminary genome assembly was likely contaminated with the symbiotic bacteria, fungal, and bacterial contaminants. To remove possible bacterial sequences, a sequence search using Blastn of all the contigs was conducted against the NCBI nucleotide database (with *E*-value cutoff <1e−05), and taxonomy was assigned to each contig. Each raw read was mapped to contigs using Bowtie2 (Langmead and Salzberg, 2012). Finally, the assembly was decontaminated using Blobtools v1.0 (Laetsch and Blaxter, 2017), which removed all bacterial contigs. Additionally, all contigs below 500 bp were removed from the final assembly. The quality of the draft assembly was determined by Quast (Gurevich et al., 2013). The draft genome presented here of *S. diaprepesi* contains 118 MB distributed among 35,545 contigs with contigs N50s of 11,474 bp and GC 45.01% with the longest contigs of 1,706,490 bp. There were zero N’s per 100 kbp within this assembly. We assessed the genome for completeness using BUSCO (Simão et al., 2015). A total of 982 BUSCOs in the Nematoda dataset were used, and our draft genome of *S. diaprepesi* had a complete BUSCO score of 85%. Most of these genes are single-copy loci at 79.6%, with 5.4% complete and duplicated BUSCOs, 7.1% fragmented BUSCOs, and 7.9% missing BUSCOs. Prediction of protein-coding genes above 1,000 bp contigs was carried out by using GenMark-ES/4.33 tool (Borodovsky and McIninch, 1993), which predicted 15,094 genes (Table 1).

**Table 1.** Summary Statistics of the Assembly of *Steinernema diaprepesi*.

Assembly statistics	
Size (bp)	118,329,602
Number of contigs	35,545
Largest contigs (bp)	1,706,490
GC content (%)	45.01
N50 value (bp)	11,474
N’s per 100 kbp	0
Number of predicted genes	15,094
Complete BUSCOs	835 (85%)
Complete and single-copy BUSCOs	782 (79.6%)
Complete and duplicated BUSCOs	53 (5.4%)
Fragmented BUSCOs	70 (7.1%)
Missing BUSCOs	77 (7.9%)

Due to the draft nature of this genome, it is incomplete, and we expect to see genome size variation between different isolates of the same nematode as *Steinernema feltiae* 82.5 Mb (Dillman et al., 2015) and 121.6 Mb (Fu et al., 2020). To confirm the genome size, checking for heterozygosity among the reads or using flow cytometry could provide a more accurate estimation. In its current state, this draft genome can provide support for comparative genomics of *Steinernema* nematodes, understand the evolution of genome network, genomic variation between different isolates, evolutionary process, and enable the functional genomics among entomopathogenic nematodes.

The Whole Genome Project of *S. diaprepesi* is deposited at GenBank under the accession number JAANPW000000000. All DNA sequence data are deposited in GenBank under Biosample No. SAMN14073714 Bio project No. PRJNA605202.

## References

[ref001] Ali, J. G. , Alborn, H. T. and Stelinski, L. L. 2010. Subterranean herbivore-induced volatiles released by citrus roots upon feeding by *Diaprepes abbreviatus* recruit entomopathogenic nematodes. Journal of Chemical Ecology 36:361–368. 20309617 10.1007/s10886-010-9773-7

[ref002] Andrews, S. 2010. “FastQC: a quality control tool for high throughput sequence data”, available at: http://www.bioinformatics.babraham.ac.uk/projects/fastqc (accessed May 30, 2020).

[ref003] Bankevich, A. , Nurk, S. , Antipov, D. , Gurevich, A. A. , Dvorkin, M. , Kulikov, A. S. , Lesin, V. M. , Nikolenko, S. I. , Pham, S. , Prjibelski, A. D. , Pyshkin, A. V. , Sirotkin, A. V. , Vyahhi, N. , Tesler, G. , Alekseyev, M. A. and Pevzner, P. A. 2012. SPAdes: a new genome assembly algorithm and its applications to single-cell sequencing. Journal of Computational Biology 19:455–477. 22506599 10.1089/cmb.2012.0021 PMC3342519

[ref004] Beavers, J. B. , McCoy, C. W. and Kaplan, D. T. 1983. Natural enemies of subterranean *Diaprepes abbreviatus* (Coleoptera: Curculionidae) Larvae in Florida1. Environmental Entomology 12:840–843.

[ref005] Bolger, A. M. , Lohse, M. and Usadel, B. 2014. Trimmomatic: a flexible trimmer for Illumina sequence data. Bioinformatics 30:2114–2120. 24695404 10.1093/bioinformatics/btu170 PMC4103590

[ref006] Bolger, M. E. , Weisshaar, B. , Scholz, U. , Stein, N. , Usadel, B. and Mayer, K. F. X. 2014. Plant genome sequencing – applications for crop improvement. Current Opinion in Biotechnology 26:31–37. 24679255 10.1016/j.copbio.2013.08.019

[ref007] Borodovsky, M. and McIninch, J. 1993. GENMARK: parallel gene recognition for both DNA strands. Computers and Chemistry 17:123–133.

[ref008] Campos-Herrera, R. , El-Borai, F. E. and Duncan, L. W. 2015. “It takes a village: Entomopathogenic nematode community structure and conservation biological control in Florida (U.S.) orchards”, in Campos-Herrera, R. (Ed.), Nematode pathogenesis of insects and other pests Springer, Cham, 329–351.

[ref009] Campos-Herrera, R. , Pathak, E. , El-Borai, F. E. , Stuart, R. J. , Gutiérrez, C. , Rodríguez-Martín, J. A. , Graham, J. H. and Duncan, L. W. 2013. Geospatial patterns of soil properties and the biological control potential of entomopathogenic nematodes in Florida citrus groves. Soil Biology and Biochemistry 66:163–174.

[ref010] Castillo, J. C. , Reynolds, S. E. and Eleftherianos, I. 2011. Insect immune responses to nematode parasites. Trends in Parasitology 27:537–547. 21982477 10.1016/j.pt.2011.09.001

[ref011] Cherry, R. , Hall, D. G. , Wilson, A. and Baucum, L. 2011. First report of damage by the Sugarcane Root Weevil *Diaprepes abbreviatus* (Coleoptera: Curculionidae) to Florida Sugarcane. Florida Entomologist 94:1063–1065.

[ref012] Dillman, A. R. , Macchietto, M. , Porter, C. F. , Rogers, A. , Williams, B. , Antoshechkin, I. , Lee, M. M. , Goodwin, Z. , Lu, X. , Lewis, E. E. , Goodrich-Blair, H. , Stock, S. P. , Adam, B. J. , Sternberg, P. W. and Mortazavi, A. 2015. Comparative genomics of *Steinernema* reveals deeply conserved gene regulatory networks. Genome Biology 16:1–21. 26392177 10.1186/s13059-015-0746-6 PMC4578762

[ref013] Donn, S. , Griffiths, B. S. , Neilson, R. and Daniell, T. J. 2008. DNA extraction from soil nematodes for multi-sample community studies. Applied Soil Ecology 38:20–26.

[ref014] Duncan, L. W. , Graham, J. H. , Dunn, D. C. , Zellers, J. , Mccoy, C. W. and Nguyen, K. 2003. Incidence of endemic entomopathogenic nematodes following application of *Steinernema riobrave* for Control of *Diaprepes abbreviatus* . Journal of Nematology 35:178–186. 19265992 PMC2620623

[ref015] Duncan, L. W. , Stuart, R. J. , El-Borai, F. E. , Campos-Herrera, R. , Pathak, E. , Giurcanu, M. and Graham, J. H. 2013. Modifying orchard planting sites conserves entomopathogenic nematodes, reduces weevil herbivory and increases citrus tree growth, survival and fruit yield. Biological Control 64:26–36.

[ref016] Fu, Z. , Li, Y. , Elling, A. A. and Snyder, W. E. 2020. A draft genome of a field-collected Steinernema feltiae strain NW. Journal of Nematology 52:1–6. 10.21307/jofnem-2020-003 PMC7265891 32180379

[ref017] Goodrich-Blair, H. and Clarke, D. J. 2007. Mutualism and pathogenesis in *Xenorhabdus* and *Photorhabdus*: two roads to the same destination. Molecular Microbiology 64:260–268. 17493120 10.1111/j.1365-2958.2007.05671.x

[ref018] Gurevich, A. , Saveliev, V. , Vyahhi, N. and Tesler, G. 2013. QUAST: quality assessment tool for genome assemblies. Bioinformatics 29:1072–1075. 23422339 10.1093/bioinformatics/btt086 PMC3624806

[ref019] Hominick, W. M. , Briscoe, B. R. , del Pino, F. G. , Heng, J. , Hunt, D. J. , Kozodoy, E. , Mracek, Z. , Nguyen, K. B. , Reid, A. P. , Spiridonov, S. , Stock, P. , Sturhan, D. , Waturu, C. and Yoshida, M. 1997. Biosystematics of entomopathogenic nematodes: current status, protocols and definitions. Journal of Helminthology 71:271–298. 9443947 10.1017/s0022149x00016096

[ref020] Laetsch, D. R. and Blaxter, M. L. 2017. BlobTools: interrogation of genome assemblies. F1000Research, 6:1287.

[ref021] Langmead, B. and Salzberg, S. L. 2012. Fast gapped-read alignment with Bowtie 2. Nature Methods 9:357–359. 22388286 10.1038/nmeth.1923 PMC3322381

[ref022] Lapointe, S. L. , Borchert, D. M. and Hall, D. G. 2007. Effect of low temperatures on mortality and oviposition in conjunction with climate mapping to predict spread of the root weevil *Diaprepes abbreviatus* and introduced natural enemies. Environmental Entomology 36:73–82. 17349119 10.1603/0046-225x(2007)36[73:eoltom]2.0.co;2

[ref023] Lu, D. , Baiocchi, T. and Dillman, A. R. 2016. Genomics of entomopathogenic nematodes and implications for pest control. Trends in Parasitology 32:588–598. 27142565 10.1016/j.pt.2016.04.008 PMC4969101

[ref024] McCoy, C. W. , Stuart, R. J. , Duncan, L. W. and Nguyen, K. 2002. Field efficacy of two commercial preparations of entomopathogenic nematodes against larvae of *Diaprepes abbreviatus* (Coleoptera: Curculionidae) in Alfisol type soil. Florida Entomologist 85:537–544.

[ref025] Nguyen, K. B. and Duncan, L. W. 2002. *Steinernema diaprepesi* n. sp. (Rhabditida: Steinernematidae), a parasite of the citrus root weevil *Diaprepes abbreviatus* (L) (Coleoptera: Curculionidae). Journal of Nematology 34:159–70. 19265926 PMC2620540

[ref026] Ogier, J. C. , Pagès, S. , Bisch, G. , Chiapello, H. , Médigue, C. , Rouy, Z. , Teyssier, C. , Vincent, S. , Tailliez, P. , Givaudan, A. and Gaudriault, S. 2014. Attenuated virulence and genomic reductive evolution in the entomopathogenic bacterial symbiont species, *Xenorhabdus poinarii* . Genome Biology and Evolution 6:1495–1513. 24904010 10.1093/gbe/evu119 PMC4079199

[ref027] Rodríguez-Leal, D. , Lemmon, Z. H. , Man, J. , Bartlett, M. E. and Lippman, Z. B. 2017. Engineering quantitative trait variation for crop improvement by genome editing. Cell 171:470–480. 28919077 10.1016/j.cell.2017.08.030

[ref028] Shapiro, D. I. , McCoy, C. W. , Fares, A. , Obreza, T. and Dou, H. 2000. Effects of soil type on virulence and persistence of entomopathogenic nematodes in relation to control of *Diaprepes abbreviatus* (Coleoptera: Curculionidae). Environmental Entomology 29:1083–1087.

[ref029] Simão, F. A. , Waterhouse, R. M. , Ioannidis, P. , Kriventseva, E. V. and Zdobnov, E. M. 2015. BUSCO: assessing genome assembly and annotation completeness with single-copy orthologs. Bioinformatics 31:3210–3212. 26059717 10.1093/bioinformatics/btv351

[ref030] Stock, S. P. and Blair, H. G. 2008. Entomopathogenic nematodes and their bacterial symbionts: the inside out of a mutualistic association. Symbiosis 46:65–75.

[ref031] Stock, S. P. , Campos-Herrera, R. , El-Borai, F. E. and Duncan, L. W. 2018. *Steinernema khuongi* n. sp. (Panagrolaimomorpha, Steinernematidae), a new entomopathogenic nematode species from Florida, USA. Journal of Helminthology 93:226–241. 29743130 10.1017/S0022149X18000081

[ref032] Stuart, R. J. , El-Borai, F. E. and Duncan, L. W. 2008. From augmentation to conservation of entomopathogenic nematodes: trophic cascades, habitat manipulation and enhanced biological control of *Diaprepes abbreviatus* root weevils in Florida citrus groves. Journal of Nematology 40:73–84. 19259523 PMC2586536

